# Construction of an Acetate Metabolic Pathway to Enhance Electron Generation of Engineered *Shewanella oneidensis*

**DOI:** 10.3389/fbioe.2021.757953

**Published:** 2021-11-19

**Authors:** Junqi Zhang, Zheng Chen, Changjiang Liu, Jianxun Li, Xingjuan An, Deguang Wu, Xi Sun, Baocai Zhang, Longping Fu, Feng Li, Hao Song

**Affiliations:** ^1^ Frontier Science Center for Synthetic Biology and Key Laboratory of Systems Bioengineering (Ministry of Education), Tianjin University, Tianjin, China; ^2^ Collaborative Innovation Center of Chemical Science and Engineering, School of Chemical Engineering and Technology, Tianjin University, Tianjin, China; ^3^ Institute of Food Science and Technology, Chinese Academy of Agricultural Sciences, Beijing, China; ^4^ Department of Brewing Engineering, Moutai Institute, Renhuai, China; ^5^ College of Biological Engineering, Tianjin Agricultural University, Tianjin, China; ^6^ College of Chemistry, Nankai University, Tianjin, China

**Keywords:** acetate, microbial fuel cell, synthetic biology, *Shewanella oneidensis*, coulombic efficiency

## Abstract

**Background:** Microbial fuel cells (MFCs) are a novel bioelectrochemical devices that can use exoelectrogens as biocatalyst to convert various organic wastes into electricity. Among them, acetate, a major component of industrial biological wastewater and by-product of lignocellulose degradation, could release eight electrons per mole when completely degraded into CO_2_ and H_2_O, which has been identified as a promising carbon source and electron donor. However, *Shewanella oneidensis* MR-1, a famous facultative anaerobic exoelectrogens, only preferentially uses lactate as carbon source and electron donor and could hardly metabolize acetate in MFCs, which greatly limited Coulombic efficiency of MFCs and the capacity of bio-catalysis.

**Results:** Here, to enable acetate as the sole carbon source and electron donor for electricity production in *S. oneidensis*, we successfully constructed three engineered *S. oneidensis* (named AceU1, AceU2, and AceU3) by assembling the succinyl-CoA:acetate CoA-transferase (SCACT) metabolism pathways, including acetate coenzyme A transferase encoded by *ato1* and *ato2* gene from *G. sulfurreducens* and citrate synthase encoded by the *gltA* gene from *S. oneidensis*, which could successfully utilize acetate as carbon source under anaerobic and aerobic conditions. Then, biochemical characterizations showed the engineered strain AceU3 generated a maximum power density of 8.3 ± 1.2 mW/m^2^ with acetate as the sole electron donor in MFCs. In addition, when further using lactate as the electron donor, the maximum power density obtained by AceU3 was 51.1 ± 3.1 mW/m^2^, which approximately 2.4-fold higher than that of wild type (WT). Besides, the Coulombic efficiency of AceU3 strain could reach 12.4% increased by 2.0-fold compared that of WT, which demonstrated that the engineered strain AceU3 can further utilize acetate as an electron donor to continuously generate electricity.

**Conclusion:** In the present study, we first rationally designed *S. oneidensis* for enhancing the electron generation by using acetate as sole carbon source and electron donor. Based on synthetic biology strategies, modular assembly of acetate metabolic pathways could be further extended to other exoelectrogens to improve the Coulombic efficiency and broaden the spectrum of available carbon sources in MFCs for bioelectricity production.

## Introduction

During the last few decades, energy exhaustion, water scarcity and environmental pollution have been among the greatest challenges of our time. Therefore, researchers are increasingly interested in the development of cost-effective methods for capturing potential energy from high-concentration saline wastewater. Bioelectrochemical systems (BESs), such as microbial electrolysis cells (Luo et al., 2011; Zhang and Angelidaki, 2014; Jafary et al., 2015) (MECs) and microbial fuel cells (Chaudhuri and Lovley, 2003; Logan, 2009; Logan and Rabaey, 2012; Zhao et al., 2020) (MFCs) are promising technologies for sustainable power generation and contaminants degradation (Xie et al., 2013; Wang et al., 2015; Butti et al., 2016; Mohan et al., 2016; Kronenberg et al., 2017; Kumar et al., 2017; Santoro et al., 2017; Zhen et al., 2017; Ilamathi and Jayapriya, 2018). In particular, MFCs can directly harvest electrical power from wastewater by employing electroactive microorganisms (Li et al., 2018a; Li et al., 2018b). Compared with traditional electrocatalysis systems, much variety of substrates which include organic acids (lactate (Chae et al., 2009), acetate (You et al., 2015), pyruvate (Pillot et al., 2020) etc.) and various carbohydrates (glucose (Chae et al., 2009), starch (Han et al., 2020) etc.) in wastewater and marine sediment could be used as electron donors/acceptors for BESs due to diversified electrochemically active bacteria (EAB). As a major component of industrial biological wastewater and lignocellulosic biomass hydrolysate, acetate has been paid great attention to bio-manufacturing and has a strong potential to compete with sugar-based carbon source (Lim et al., 2018; Novak and Pflügl, 2018). However, utilization of acetate is limited in many EAB by some biological factors which like lack of uptake transporters (Enerson and Drewes, 2003; Gimenez et al., 2003; Morris and Felmlee, 2008), energetic requirements for transport (Lin et al., 2006), or the availability of enzymes to oxidize substrates (Brown et al., 1977; Kumari et al., 1995; Dittrich et al., 2005; Shi et al., 2005).

*Shewanella oneidensis* MR-1, one of the most widely studied dissimilatory metal-reducing bacterium, can conduct extracellular electrons transfer (EET) through its electroactive biofilm. It has been extensively studied for dissecting inward EET mechanism (Pirbadian et al., 2014; Kumar et al., 2016; Xiao et al., 2017; Light et al., 2018), developing gene editing tools (Yu et al., 2011; Liu et al., 2015; Yang et al., 2015), exploring novel technologies for polymer and nanoparticle synthesis (Yong et al., 2012; Yong et al., 2014; Yu et al., 2015), bioremediations of toxic metals (Vikrant et al., 2018), and electro-fermentations (Logan et al., 2008; Varrone et al., 2014; Kitching et al., 2017) in recent decades. However, *S. oneidensis* MR-1 has great limitation in substrate utilization, which hinder its practical applications in BESs. Lactate, as a preferential electron donor, could only be consumed and converted to acetate, which could not to be metabolized to CO_2_ and H_2_O under anaerobic conditions in MFCs, resulting insufficient utilization of lactate and leaving eight electrons in acetate. Even to enhance the efficiency of electron donor utilization, constructing a synthetic microbial consortium does not completely release electrons. Thus, the incomplete utilization of electron donors of *S. oneidensis* extremely decreases the Coulombic efficiency of MFCs and restricts the development and applications of various BES.

In microorganisms, acetate is usually metabolized to acetyl-CoA *via* three pathways catalyzed by: 1) acetate kinase phosphotransacetylase (ACKA-PTA) (Rose et al., 1954; Dittrich et al., 2005; Shi et al., 2005) encoded by *ackA* and *pta*, 2) acetyl-CoA synthetase (ACS) (Jogl and Tong, 2004) encoded by *acs* or 3) acetate coenzyme A transferase, encoded by *ato1* and *ato2* gene, respectively. Both of ACKA-PTA and ACS pathway need to consume ATP to drive acetate utilization, but acetate could be efficiently converted to acetyl-CoA by assistance of succinyl-CoA without ATP consumption in the third pathway in *Geobacter sulfurreducens*. ACKA-PTA pathway in *S. oneidensis* could metabolize acetate under aerobic conditions. However, acetate is hardly utilized for *S. oneidensis* due to insufficient energetic requirements by the interruption of tricarboxylic acid (TCA) cycle under anaerobic conditions. Thus, the crucial problem is how to broaden and strengthen lactate utilization efficiency of *S. oneidensis* to thoroughly release electrons.

Recently, many strategies were used for improving bioelectricity production and Coulombic efficiency in *Shewanella*-inoculated MFCs that focus on increasing intracellular electrons generation and enhancing the extracellular electrons transfer rate of exoelectrogens (Li et al., 2019; Zhang et al., 2019). For example, redirecting metabolic flux towards NAD^+^ biosynthesis resulted more electrons from the increased oxidation of electron donor to EET pathways of *S. oneidensis* (Li et al., 2018c; Li et al., 2018d). Furthermore, enhancing flavins biosynthesis and transportation in a hydrophobic chassis of *S. oneidensis* could significantly boost its EET rate and performance (Lin et al., 2018). However, increasing EET rate and Coulombic efficiency via broadening substrate spectrum and enhancing utilization rate, which are the rate-limiting steps of whole bioelectricity transfer process, have been largely neglected in the past.

**FIGURE 1:**
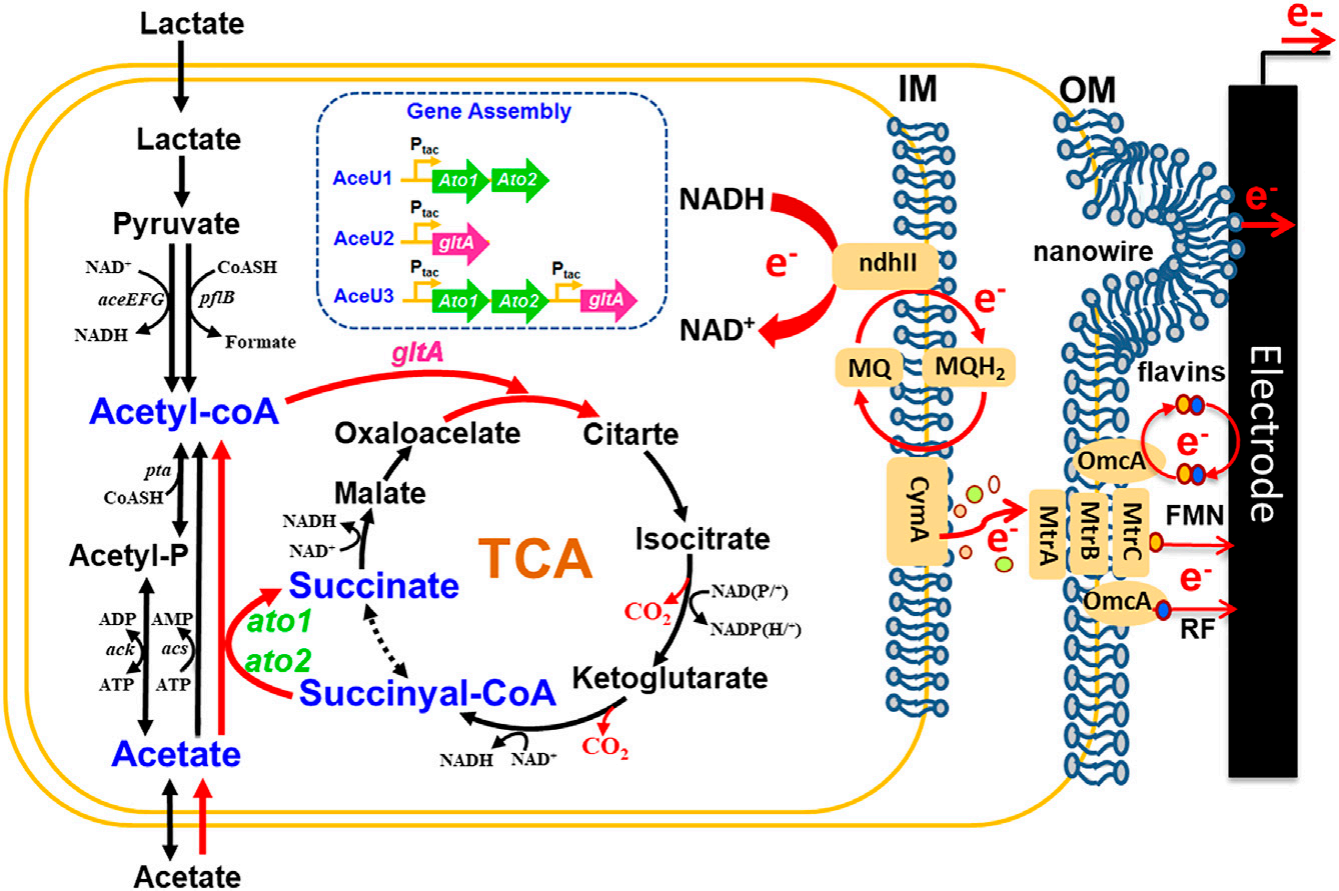
Construction of engineered *S. oneidensis* strains to enable acetate utilization and electricity generation based on synthetic biology strategies. A unique acetate utilization way constructing by *ato1* and *ato2* (the genes encoding succinyl: acetate coenzyme A transferase from *G. sulfurreducens* and *gltA* (the gene encoding citrate synthase) from *S. oneidensis*, which is free of energy-consuming and restores vitality of TCA in anaerobic conditions.

Herein, to enable *S. oneidensis* to completely utilize acetate for the bioelectricity production in MFCs under anaerobic condition, we successfully constructed engineered *S. oneidensis* by assembling the succinyl-CoA:acetate CoA-transferase (SCACT) metabolism pathway (Figure 1). At the beginning, to broaden the substrate spectrum of *S. oneidensis*, acetate coenzyme A transferase encoded by *ato1* and *ato2* gene from *G. sulfurreducens* was heterologously expressed in *S. oneidensis,* which could catalyse acetate to acetyl-CoA without ATP consumption. Then, to further accelerate the acetate assimilation rate, we also overexpressed a citrate synthase encoded by the *gltA* gene in engineered *S. oneidensis*. Finally, an engineered strain was constructed and its ability to generate electricity using acetate or lactate was compared with wild-type strain. To the best of our knowledge, this is the first report on the rationally designed *Shewanella* that could use acetate as the carbon source and electron donor to produce electricity. Furthermore, this engineering strategy also offered the possibility of other exoelectrogens to broaden the spectrum of available carbon sources and improve the Coulombic efficiency in MFCs.

## Results and Discussion

### Acetate Metabolic Pathway Construction for *S. oneidensis Via* Synthetic Biology Strategy

In *S. oneidensis*, acetate is usually converted to acetyl coenzyme A *via* ACS and ACKA/PTA pathways under aerobic conditions, and then enters the main metabolic pathway (Figure 1). However, under anaerobic condition in MFCs, both ACS and ACKA/PTA pathways have remarkably limited capacities for acetate assimilation and large accumulation of acetate can cause disturbances of intracellular pH homeostasis. Conversely, SCACT from *G. sulfurreducens* is a new pathway of acetate metabolism that enables acetate to obtain CoA directly from succinate-CoA to generate acetyl-CoA without consuming ATP and converts succinyl-CoA to succinate accelerating the TCA cycle (Ueki and Zhou, 2021). To realize *S. oneidensis* to continually utilize acetate as sole carbon source for the bioelectricity production in MFCs, we firstly adopted the synthetic biology strategies to heterologously express the acetate coenzyme A transferase encoded by *ato1* and *ato2* gene from *G. sulfurreducens* to catalyse acetate converted in to acetyl-CoA, which not only enhanced the assimilation capacity of acetate without energy consumption, but also accelerated the conversion of succinate-CoA to succinate of the TCA cycle. And then to further improve the flux of acetyl-CoA into the TCA cycle, a citrate synthase encoded by the *gltA* gene from *S. oneidensis* was further overexpressed to avoid excessive accumulation of acetyl-CoA, thus constructing acetate-fed *S. oneidensis* from reconstructing the SCACT pathway and enhancing the rate of acetate utilization.

The multi-module gene assembly was carried out as previously reported in *S. oneidensis*, a Biobrick^TM^ compatible vector named pYYDT was inductively expressed by kanamycin (Yang et al., 2015). Furthermore, to enhance codon fitness, we used *in vitro* chemical synthesis of codon-optimized genes, rather than direct molecular cloning from other bacteria. We finally constructed engineered strains AceU1 (including *ato1* and *ato2* genes for succinyl-CoA:acetate CoA-transferase), AceU2 (including *gltA* gene for the citrate synthase) and AceU3 (including *ato1*, *ato2* and *gltA* gene) for further improving the Coulombic efficiency of classical lactate fed MFCs, plasmid construction as shown in Supplementary Figure S1. Expression level of mRNA in each of these strains was quantified by real-time PCR (RT-PCR). Under the conditions of adding 0.75 mM IPTG as inducer, the transcriptional expression levels of *ato1*, *ato2* and *gltA* were 5.0, 4.1 and 5.1 folds higher than the normalization gene, respectively (Supplementary Figure S2). This result indicated that the three target genes could be well expressed in AceUs.

### Characterization of Acetate Utilization and Cell Growth of Engineered *S. oneidensis*

The cell growth activity and acetate consumption of wild-type (WT, harboring the empty vector pYYDT) and three engineering *S. oneidensis* strains AceU1, AceU2 and AceU3, were cultured in SBM medium added with 10 mM acetate as sole carbon source under aerobic and anaerobic conditions, respectively.

**FIGURE 2:**
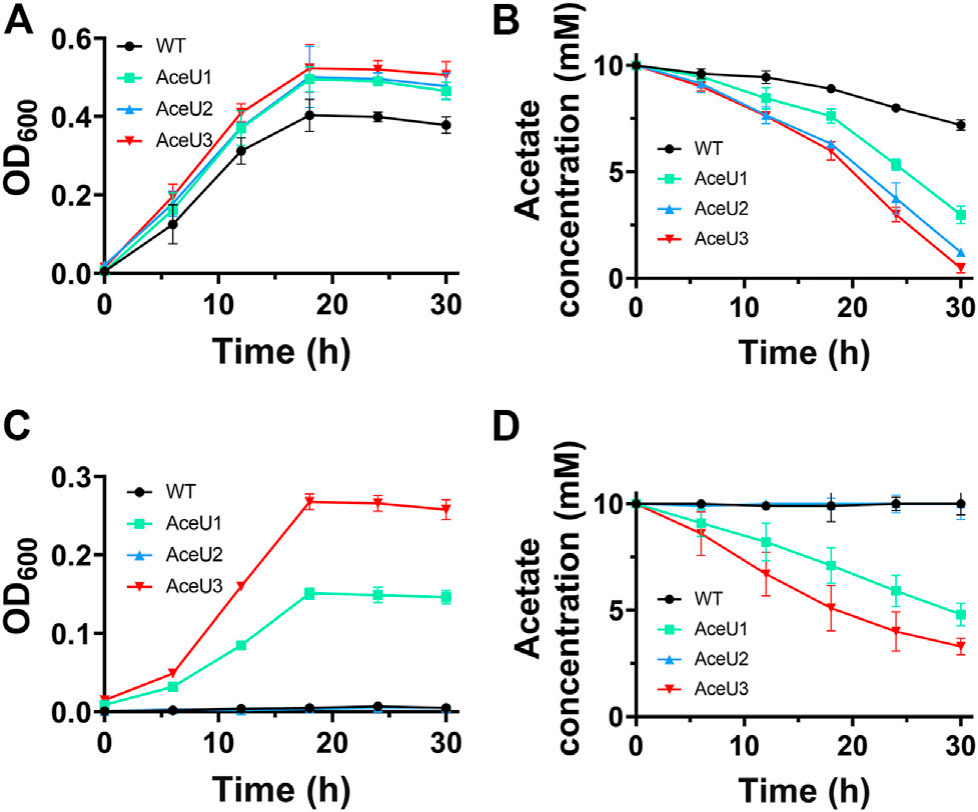
Growth curves and acetate consumption of the WT and the recombinant *S. oneidensis* strains under aerobic or anaerobic conditions. **(A)** Aerobic growth curve (OD_600_ ∼ *t*) in SBM supplemented with 10 mM acetate. **(B)** Acetate consumption under aerobic condition. **(C)** Anaerobic growth curve (OD_600_ ∼ *t*) in SBM supplemented with 10 mM acetate. **(D)** Acetate consumption under anaerobic condition. The error bars were calculated from triplicate experiments.

Under aerobic condition, the growth curves of these engineered strains demonstrate that AceU1, AceU2 and AceU3 have better ability to grow in SBM medium with acetate as the sole carbon source than the WT (Figure 2A) Additionally, the engineering strain AceU3 showed a higher growth biomass than AceU1 and AceU2. The acetate consumption rate of engineered strains AceU3 is 0.40 mM/h, which is faster than that of AceU1 and AceU2 (0.25 mM/h and 0.30 mM/h) (Figure 2B). This indicated that the constructed acetate metabolic pathway has a positive effect on cell growth under aerobic conditions.

The respiratory of WT and engineered strains were also determined under anaerobic condition with acetate as unique carbon source. Unlike aerobic condition, WT strain was barely able to use acetate while engineered strains had significant growth advantages, indicated the substrate spectrum of *S. oneidensis* had been broadened. Growth rates of engineered strains AceU1 and AceU3 were similar to aerobic condition (Figure 2C). Acetate was consumed by AceU3 at a rate of ∼0.23 mM/h, which was faster than that of AceU1 and AceU2 (∼0.17 mM/h and ∼0.0022 mM/h) (Figure 2D). These results showed that the SCACT pathway is able to convert acetate to acetyl-CoA in one step with less energy consumption compared with the native ACS and ACKA/PTA pathways. Moreover, the AceU2 showed significantly different of acetate consumption rate under both culture conditions, which is 0.30 mM/h and 0.0022 mM/h, respectively. This result further suggested that inadequate downstream carbon flux of acetyl-CoA was a major limitation of acetate metabolism under aerobic condition, whereas the limited synthesis of ATP was insufficient to supply acetate metabolic equivalents under anaerobic condition, which limiting the ability to utilize acetate. In addition, the comparison of the three engineered strains showed that AceU3 had the highest capacity for acetate utilization, indicating that combination of reconstitution of the SCACT pathway and expression of *gltA* to enhance acetyl-coA conversion can accelerate the utilization of acetate. In conclusion, we broadened the substrate spectrum of *S. oneidensis* with enhanced acetate assimilation and tolerance capacity.

### Microbial fuel cells Performance Analysis With Acetate as Sole Electron Donor

Under anaerobic condition, Dual-chamber MFCs were used to evaluate the electrochemical performance of engineered strains AceU1, AceU2, and AceU3 using acetate as the sole electron donor or carbon sources. The engineered strains and WT were inoculated into anodic chamber of MFCs, respectively. And output voltages were real-time recorded every 30 min with a data acquisition system.

**FIGURE 3:**
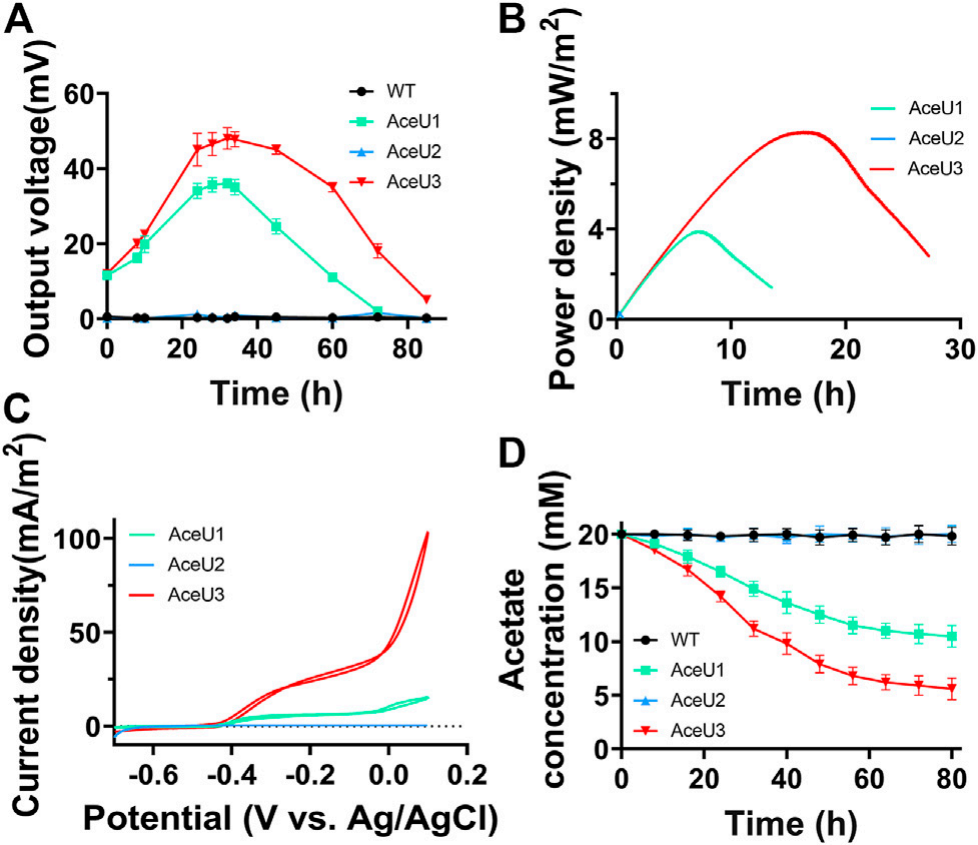
Electrochemical analysis of the electrical production properties of WT and engineered *S. oneidensis* strains (AceU1, AceU2 and AceU3) with acetate as carbon sources. **(A)** MFCs discharge curve of four strains WT, AceU1, AceU2 and AceU3, using acetate as carbon source. 20 mM acetate was added at the initiation of MFC operations. **(B)** MFCs power density output curves obtained by linear sweep voltammetry (LSV) with a slow scan rate of 0.1 mV/s. **(C)** Cyclic voltammetry (CV) analysis of four strains. **(D)** The acetate consumption curve in MFC.

Initially, 20 mM acetate was added as electron donor to evaluate the power output capacity of each engineered *S. oneidensis* strain in MFCs (Figure 3A). The WT and AceU2 strains could barely generate any voltage output with acetate as sole carbon source, which illustrated that WT and AceU2 strains could not utilize acetate. Whereas the engineered strains AceU1 and AceU3 could generate a maximum output voltage of∼35 ± 3.5 mV and∼50.2 ± 1.3 mV (*n* = 3), suggesting that genetically programmed SCACT pathway genes and citrate synthase genes could activate acetate metabolism and enabled acetate as the sole electron donor for electricity production. The electron transfer efficiency also was analyzed by linear sweep voltammetry (LSV) and cyclic voltammetry (CV) as shown in Figures 3B,C. The AceU3 strain had the best electrochemical performance with a peak power density of 8.3 ± 1.2 mW/m^2^ (Figure 3B). Among the three engineered strains, AceU3 is the combination of AceU1 and AceU2 functions, which showed better electrochemical properties. Current density of AceU3 was obviously improved compared to other strains, indicating a higher electron transfer efficiency (Figure 3C). Meanwhile, there were typical redox peaks in CV curves starting around −0.4 V (vs Ag/AgCl), which belongs to flavins-mediated extracellular electron transfer. It suggested that the novel degradation pathway not only accelerated acetate assimilation, but also in some way promoted the flavin-mediated electron transfer process. As shown in Figure 3D, the acetate metabolite concentrations in the anolyte during MFC discharge were quantified to evaluate electrochemical performance. Acetate consumption of AceU3 strains was significantly better than AceU1 and AceU2, and these results were consistent with the power generation. Thus, it proved the development of bioelectricity performances of engineered strains was brought out by the accelerated acetate utilization rate.

### Electrochemical Properties Analysis With Lactate as Sole Electron Donor

**FIGURE 4:**
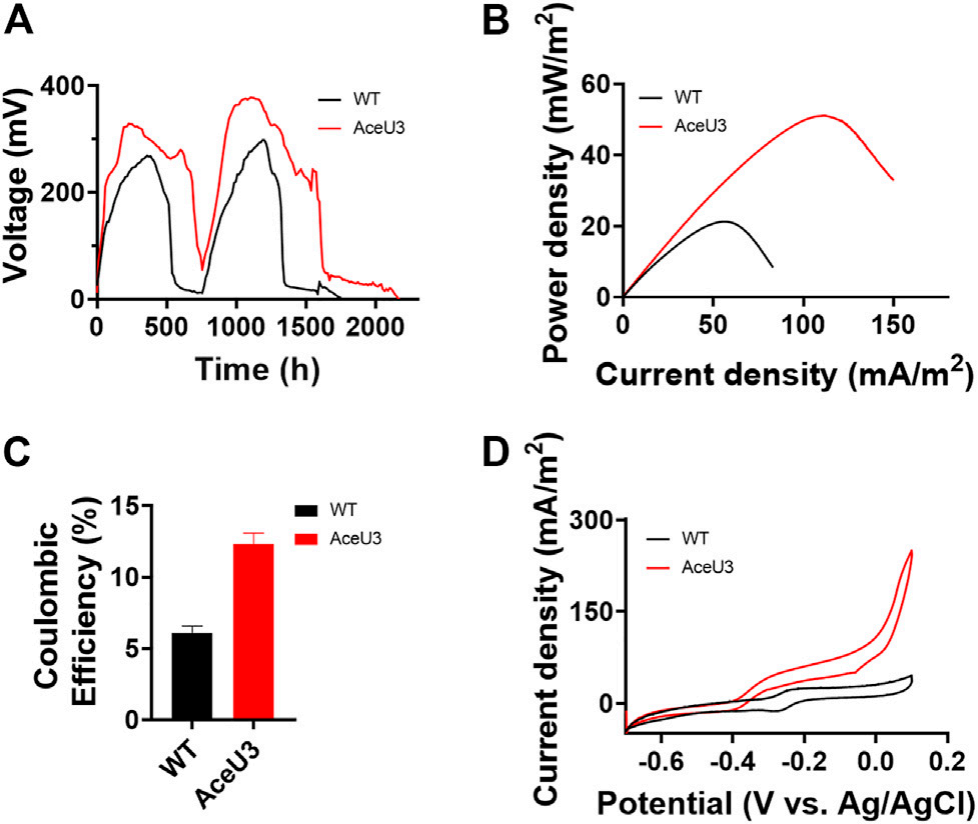
Electrochemical performances analysis of WT and ET *S. oneidensis* in MFCs with lactate as carbon source. **(A)** Output voltage curves changed a part of anolyte with fresh anolyte when output voltage was completely declined. Catholyte was changed whenever needed. **(B)** MFCs power density output curves obtained by linear sweep voltammetry (LSV) with a fixed scan rate of 0.1 mV/s. **(C)** Coulombic efficiency of MFCs which inoculated WT and AceU3. **(D)** Cyclic voltammetry (CV) analysis of four strains. All error bars were calculated from triplicate experiments.

It is well known that acetate is a secondary metabolite produced by incomplete oxidation of lactate which is one of the most favorable carbon sources under anaerobic conditions in *S. oneidensis*. Although we have successfully constructed engineered strains using acetate as a carbon source and electron donor, it is unknown whether the electrons in lactate can be fully obtained and then enhanced coulomb efficiency when using lactate as a substrate, which contains a higher number of electrons. To verify the changes of lactate utilization capacity and coulomb efficiency of the engineered strain, the multiple cycles of voltage output of AceU3 were measured in MFCs to assess multicycle operation (Figure 4A). After 760 h when output voltage was firstly decreased to baseline level, another 20 mM lactate was added to anode chamber. Both MFCs of WT and AceU3 showed stable electricity generation during two consecutively discharged cycles, but AceU3 duration in steady state was longer than that of WT in each cycle. Moreover, the output voltage of AceU3 (328.2 ± 6.0 mV and 375.4 ± 4.2 mV) in each cycle was higher than of that of WT (272.1 ± 5.5 mV and 298 ± 4.6 mV), indicating that some new redox reactions joined into the system and changed to anodic electrodynamic potential providing a more powerful driving force. Interestingly, the output voltage of AceU3 in second cycle was obviously higher than the first cycle, indicated that electroactive biofilms were formed on the surface of the electrodes during the first cycle. It suggested that the novel acetate metabolism pathway could promote the formation of electroactive biofilm. Another evidence for the engineered metabolism system facilitating electron transfer is the performance of power density. The power output showed that AceU3 had the highest power density (51.0 ± 3.1 mW/m^2^), which is 2.4 times higher than that of WT (21.1 ± 2.4 mW/m^2^) (Figure 4B).

**FIGURE 5:**
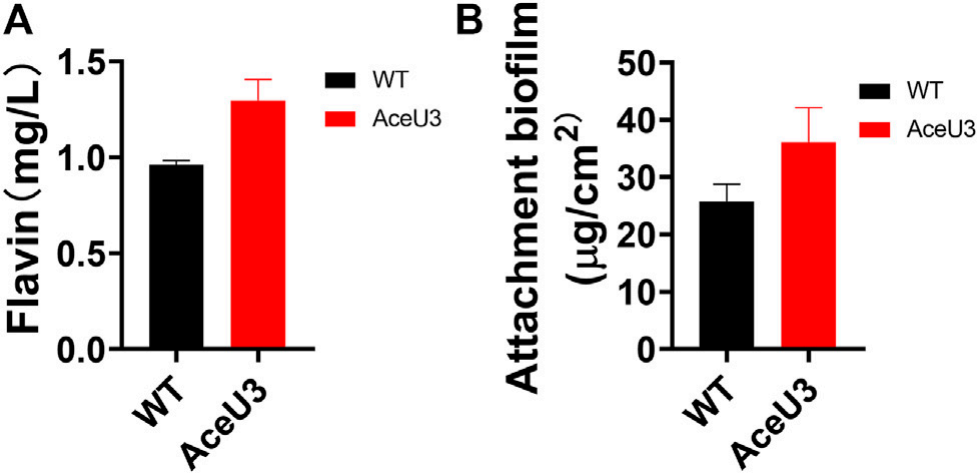
Biochemical analyses of the engineered *S. oneidensis* and WT. **(A)** Determination of flavin concentration in the anolytes of MFCs, and **(B)** the attached biomass of each strain on anode surfaces. All error bars were calculated from triplicate experiments.

Furthermore, the Coulombic efficiency was also used to calculate the Coulombic rate between the actual recovered and the total amount in the substrate. And the Coulombic efficiency calculations showed that AceU3 had the highest Coulombic efficiency in MFC (12.4%) which was 2 times that of the WT strain (Figure 4C). It illustrated that the novel pathway could enhance lactate utilization efficiency and avoid more electrons lost in other processes. Besides, the dropping slope of polarization curves obtained from the strain AceU3 was smaller than one obtained from WT, implying that the internal charge transfer resistance of the MFC inoculated with AceU3 was relatively smaller (Supplementary Figure S3). Furthermore, to further study the EET efficiency of engineered strain AceU3, the cyclic voltammetry (CV) at 1 mV/s was applied to reveal the redox reaction kinetics. As shown in Figure 4D, there were typical redox peaks of flavin in the CV curves at ∼ −0.4V (vs Ag/AgCl), which indicated that the EET of AceU3 is similar with WT but with higher current density than that of WT. Meanwhile, biochemical characterization showed that the biosynthesis of flavin was increased in AceU3, confirming to the analysis results of CV (Figure 5A). In addition, the amount of attached biomass was assessed, and the formation of biofilm on the electrode surface was observed by confocal microscope (Figure 5B; Supplementary Figure S4). This result indicated that engineered strain AceU3 possessed a stronger drive to fully assimilate lactate, and accelerated the TCA cycle. Furthermore, it also illustrated that enhanced acetate consumption could enhance biofilm formation and improve cellular activity. It is speculated that this may be due to the enhanced synthesis and regulation of c-di-GMP by acetate metabolism, which could promote biofilm formation and EET in *Shewanella* (Mukherjee et al., 2020; Ng et al., 2020). In addition, the efficient biofilm formation on anodes in the engineered *S. oneidensis* strains enabled an accumulation of flavin which eventually enhanced EET efficiency and electricity generation.

## Conclusion

In the present study, we first rationally designed *S. oneidensis* for enhancing the electron generation by using acetate as sole carbon source and electron donor. To enable *S. oneidensis* to continually utilize acetate as the sole carbon source for bioelectricity generation in MFCs after lactate was completely consumed, we rationally constructed engineered *S. oneidensis* (namely AceUs) by heterologously overexpressing the citrate synthase (encoding by the *gltA* gene) and acetyl-CoA transferase (encoding by *ato1* and *ato2* gene) from *Geobacter sulfurreducens*. For the first time we broadened the substrate spectrum of *S. oneidensis* that could degrade acetate in MFCs and present an accelerated electricity transfer rate. Moreover, when using lactate as sole electron donor for MFCs, the maximum power density of AceU3 strain could reach 51.1 mW/m^2^, which was 2.4 times than that of WT strain. In addition, the Coulombic efficiency of AceU3 strain could reach 12.4%, surprisingly higher than that of the control strain (6.1%). Our work rationally engineered the metabolism of acetate after lactate was fully consumed and significantly enlarged the spectrum of carbon sources that could be taken by *S. oneidensis*.

## Methods

### *In vitro* Gene Synthesis

The *gltA* gene encoding the citrate synthase, *ato1* and *ato2* genes encoding the acetyl-CoA transferase enzymes originated from *Geobacter sulfurreducens* PCA were identified in the NCBI database. Subsequently, the gene codon sequences were optimized for *S. oneidensis* MR-1 in Java codon adaption tool (JCAT) in order to replace rare codons of tRNAs to ensure the translation (Supplementary Table S1). The restriction enzyme sites of *EcoRI*, *XbaI*, *SpeI* and *SbfI* were avoided in the optimized sequences.

### Strain Construction and Transformation and Culture

The plasmid construction was performed in *E. coli* Trans T1 that was cultured in the LB (Luria–Bertani) medium at 37°C with 220 rpm. The gene biobrick including *gltA*, *ato1* and *ato2* was inserted into the vector pYYDT to form the expression plasmid pYYDT-AceUs (see Supplementary Table S2 for primer sequences) by BioBrick^®^ Assembly Kit (New England BioLabs inc., United States). The pYYDT- AceUs was firstly transformed into the plasmid donor strain *E. coli* WM3064 (a dap auxotroph) which was needed to add 100 μ g/ml 2,6-diaminopimelic acid (DAP) for the growth, then transferred into *S. oneidensis* by conjugation. Whenever needed, 50 µg/ml kanamycin was added in the culture medium. All the strains and plasmids are listed in Table 1.

**TABLE 1:** Strains and plasmids used in this study.

Strains or plasmids	Feature (s)	Source
Strains		
*S. oneidensis*		
MR-1 (wild type)	Parent strain	Our lab
AceU1	Carrying pYYDT—AceU1	This study
AceU2	Carrying pYYDT—AceU2	This study
AceU3	Carrying pYYDT—AceU3	This study
*E. coli*		
Trans T1	F- φ 80 (*lac*Z) ΔM15Δ*lac*X74*hsdR*	Transgen Biotech
	(rk^−^, m_k_^+^) Δ*rec*A1398*end*A1tonA	
WM3064	A dap auxotroph *E. coli*	Our lab
Plasmids		
pYYDT	5.9 kb; Km^r^; *lacZ*	Our lab
pYYDT-AceU1	Plasmid with the *Ato1* and *Ato2* genes inserted	This study
pYYDT-AceU2	Plasmid with the *gltA* gene inserted	This study
pYYDT-AceU3	Plasmid with the *gltA*, *Ato1* and *Ato2* genes inserted	This study

### The Expression of Target Genes Detected by Quantitative Real-Time Reverse Transcription Polymerase Chain Reaction

Quantitative real-time reverse transcription polymerase chain reaction (qRT-PCR) can be used to analyze the expression of genes by quantifying the genes cDNA abundance. To analyze the expression of *gltA*, *ato1* and *ato2* genes, the recombinant *Shewanella* was induced by 0.75 mM IPTG. When in mid-log-phase cultures, total RNA was isolated by a bacterium total RNA extraction kit (APEXBIO, China). Then, the GoScript reverse transcription system (Promega, WI, United States) was used to synthesize the cDNA. Gene *gyrB* was used as normalization. Quantitative analyses of target gene expression were performed using SsoAdvanced SYBR Green supermix (Bio-Rad, CA, United States) (Cao et al., 2017). Samples were tested in triplicate using the listed primers (Supplementary Table S2).

### Evaluation Cell Growth of Wild Type, AceU Strains and Quantification of Acetate Consumption

To determine situation of cell growth, 2 ml culture suspension of the wild-type (WT) or engineered acetate-utilizing *S. oneidensis* strain was inoculated into 100 ml SBM medium. The cell cultures were incubated at 30°C 200 rpm, and samples were withdrawn periodically for the determination of cell density (optical density at 600 nm, i.e. *OD*_600_), and *OD*_600_ was measured by ultraviolet and visible spectrophotometer (TU-1810, Beijing, China) (Li et al., 2017). Both WT and AceU strain were cultured in 10 ml LB broth overnight at 30°C 200 rpm. Then, 3% inoculation amount of each suspension was transferred to SBM (pH 7.2) supplemented with 10 mM acetate as substrates. Whenever needed, 50 μg/ml kanamycin, 40 mM Fumarate and 0.75 mM IPTG were supplemented. The metabolites in the shake flasks were analyzed by a high-performance liquid chromatography (HPLC) system, which was equipped with a UV detector. All fermentation samples and standard solutions were pretreated by a 0.22 μm filter before HPLC testing. Acetate concentration was analyzed using HPLC with an Aminex HPX-87H column (Bio-Rad) at 65°C, using 5 mM H_2_SO_4_ as eluent, at a flow rate of 0.6 ml/min by UV spectrophotometer at 210 nm.

### Microbial Fuel Cells Setup

The WT strain and AceUs strain (harboring the empty vector pYYDT and pYYDT-AceU, respectively) from −80°C freezer stock were inoculated into 3 ml LB broth supplemented with 50 μg/ml kanamycin shaking at 30°C 200 rpm overnight. Then, 2 ml suspension was transferred to 100 ml LB broth with 50 μg/ml kanamycin and 0.75 mM IPTG as an inducer. When cultured around 12 h, the suspension was subsequently centrifuged 5,000 rpm for 10 min at 4°C. Finally, the cell pellets were adjusted to *OD*_600_ 0.5 and dispersed into 140 ml anolyte. Carbon cloth was used as the electrodes for anode (2.5 cm × 2.5 cm) and cathode (2.5 cm × 3 cm). The dual-chamber MFCs (140 ml) were separated by Nafion 117 membrane were separated by Nafion 117 membrane, which was pretreated in 1 M HCl overnight, and washed three times with sterile distilled water before MFC setup. The anolyte consisted of 95% M9 buffer supplemented with 20 mM sodium lactate and 5% (v/v) LB broth. The cathodic electrolyte was made of 50 mM K_3_[Fe(CN)_6_], 50 mM KH_2_PO_4_ and 50 mM K_2_HPO_4_. The dual-chamber MFCs were separated by Nafion 117 membrane, which was pretreated in 1 M HCl for overnight, and kept in sterile distilled water before H-cell MFC setup. The MFCs across 2 kΩ resistors were incubated at 30°C in biochemical incubator, and the output voltages were recorded by a digital multimeter (DT9205A).

### The Calculation of Coulombic Efficiency

The total coulombs, where *n*_*x*_ is the number of moles of substrate, *b*_*x*_ is the moles of electrons per mole of substrate, and *F* is Faraday’s constant. The general procedure of lactate and acetate completely degradation can be described as:

$$C_{3}H_{6}O_{3}\mathrm{+3}O_{2}\to 3CO_{2}\mathrm{+3}H_{2}O$$ (1)

$$C_{2}H_{4}O_{2}\mathrm{+2}O_{2}\to 2CO_{2}\mathrm{+2}H_{2}O$$ (2)

which means each mole of lactate and acetate could contribute 12 and 8 mol electrons ( $b_{1}=12$ and $b_{a}=8$ ) respectively. The Coulombs actually recovered was determined by integrating the current (*I*) over a period of batch cycle (*t*). So, the Coulombic efficiency can be evaluated over a period of time as:

$$C_{E}=\frac{M_{S}\int_{0}^{t_{b}}I\;dt}{Fb_{ES}V_{An}\Delta C}=\frac{M_{S}It_{b}}{Fb_{ES}V_{An}\Delta C}$$ (3)

where *M*_*S*_ (g/mol) is the molecular weight of the substrate, *F* is Faraday’s constant (98,485 C/mol of electrons), *C* is the symbol of Coulomb, *I* (A) is the current, *t*_*b*_ (s) is the time period of a batch cycle, *b*_*ES*_ is the stoichiometric number of moles of electrons produced per mole of substrate, *V*_*An*_ (L) is the volume of liquid in the anode compartment, and Δ*c* (g/L) is the substrate concentration change over the bath cycle time (Yan et al., 2015; Li et al., 2018e).

### Electrochemical Analyses

Electrochemical analysis is carried out at the steady state of the MFCs when the output voltage of the MFCs is at its maximum. Cyclic voltammetry (CV) analysis was conducted on a three-electrode mode with a scan rate of 1 mV/s, in which the Ag/AgCl as reference electrode by a CHI1000C multichannel electrochemical workstation (CH Instrument, Shanghai, China). To obtain the polarization curves, linear sweep voltammetry (LSV) analysis with a slow scan rate of 0.1 mV/s was conducted on a two-electrode mode by an electrochemical workstation CHI1000C (CH Instrument, Shanghai, China).

### Quantification of Riboflavin and Electrode Attachment Biomass

For determination of riboflavin content in MFC, the 5 ml of anode solution was collected, then centrifuged at 1,200 rpm/min for 5 min and filtered through a 0.22 μm filter membrane to remove bacteria. All standard solutions and samples were determined by HPLC with reverse-phase C18 column (10 cm × 2.1 mm, 5 μm) (Yang et al., 2015). Anodic attached biofilm measurement, the anode carbon cloth was collected and placed in a 50 ml test tube. Allow the cells to lyse and assay using the pierce^TM^ BCA protein assay kit (made by Thermo) (Li et al., 2017). Biofilms biovolume and thickness in the anode carbon cloth were monitored using a CLSM (Nikon, Ti2-E full electric inverted microscope) (Mukherjee et al., 2018).

## Data Availability

The raw data supporting the conclusion of this article will be made available by the authors, without undue reservation.

## Abbreviations

BES, bioelectrochemical systems; CV, cyclic voltammetry; ET, engineered type; EET, extracellular electron transfer; LSV, linear sweep voltammetry; MECs, microbial electrolytic cells; MFCs, microbial fuel cells; WT, wild type; TCA, tricarboxylic acid cycle.
