# Supplementary material for: Construction of an Acetate Metabolic Pathway to Enhance Electron Generation of Engineered Shewanella oneidensis
Source: Front Bioeng Biotechnol. 2021 Nov 19;9:757953. doi: 10.3389/fbioe.2021.757953 (PMC8640130; doi:10.3389/fbioe.2021.757953)
Supplement: Supplementary file 1 [file DataSheet1.docx]

**Construction of an acetate metabolic pathway to enhance electron generation of engineered *Shewanella oneidensis***

Junqi Zhang^1,2,‡^, Zheng Chen^1,2,‡^, Changjiang Liu^1,2,‡^, Jianxun Li^3^, Xingjuan An^1,2^, Deguang Wu^4^, Xi Sun^5^, Baocai Zhang^1,2^, Longping Fu^6^, Feng Li^1,2,*^, Hao Song^1,2,*^

^1^ Frontier Science Center for Synthetic Biology and Key Laboratory of Systems Bioengineering (Ministry of Education), Tianjin University, Tianjin, 300072, China

^2^ Collaborative Innovation Center of Chemical Science and Engineering (Tianjin), School of Chemical Engineering and Technology, Tianjin University, Tianjin, 300072, China

^3^ Institute of Food Science and Technology, Chinese Academy of Agricultural Sciences, Beijing 100093, P. R. China

^4^ Department of Brewing Engineering, Moutai institute, Luban Ave, Renhuai, 564507, Guizhou, China

^5^ College of Biological Engineering, Tianjin Agricultural University, Tianjin 300384, PR China.

^6^ College of Chemistry, Nankai University, Tianjin 300071, P. R. China.

* Corresponding author: F Li, E-mail: messilifeng@163.com; H. Song, E-mail: hsong@tju.edu.cn

‡ Equal contribution.


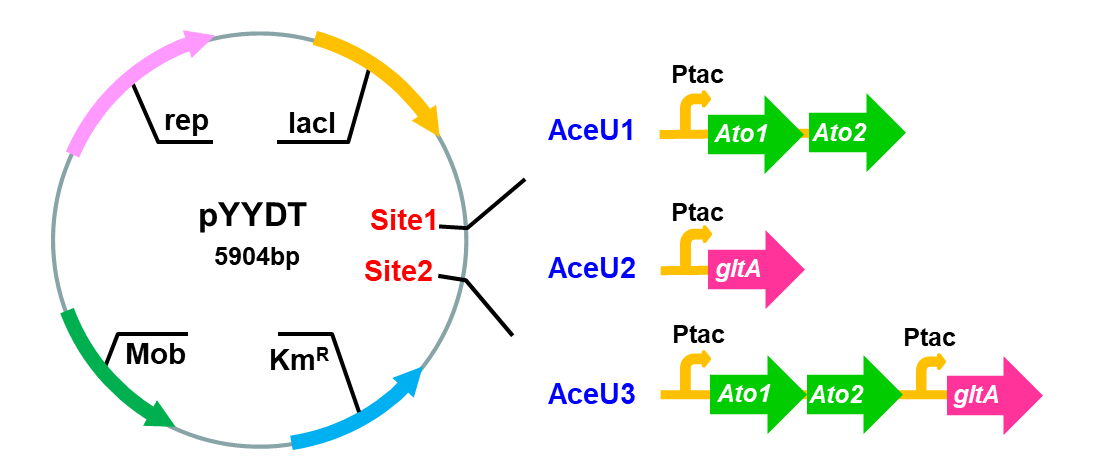


**Figure S1**. Schematic of the plasmid maps of *gltA*, *ato1* and *ato2* genes expressing vectors. Three gene assemblies (plasmids), namely AceU1, AceU2 and AceU3 were synthesized for the enhanced acetate metabolism, which transformed into *S. oneidensis*, respectively, to construct three recombinant *S. oneidensis* strains.

**Figure S2**. Under the conditions of adding 0.75 mM IPTG as inducer, qRT-PCR detection showed that the transcriptional expression levels of *ato1*, *ato2* and *gltA* were 5.0, 4.1 and 5.1 times for the normalization gene, respectively.

**Figure S3**. The polarization curves of WT and engineering S. oneidensis in MFCs with lactate as electron donor.


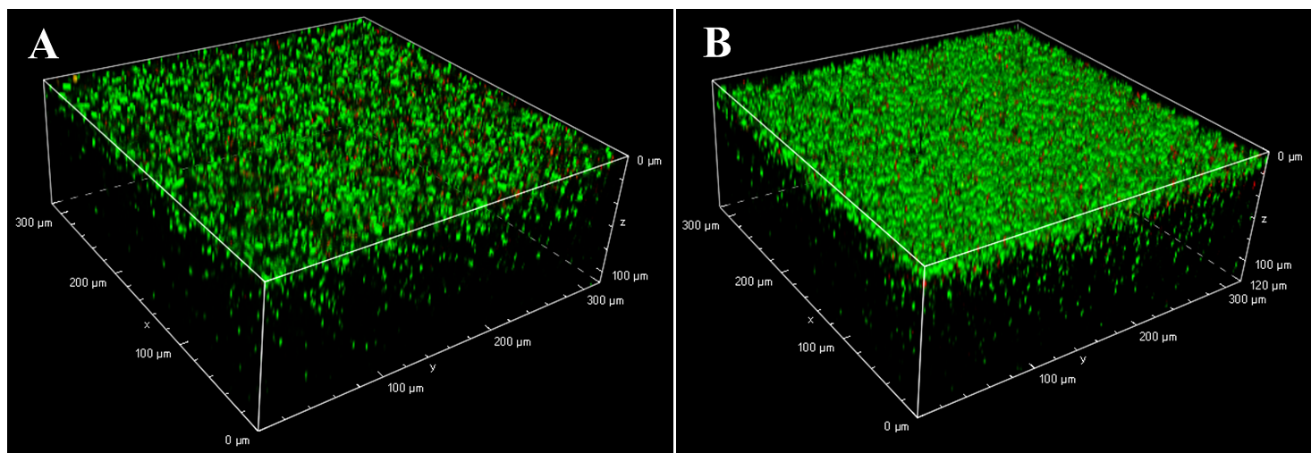


**Figure S4**. CLSM 3D images of the biofilm showing the thickness and activity. (A) Biofilm images of wild-type strains. (B) Biofilm image of the engineered strain AceU3.
